# Is Adynamic Bone Always a Disease? Lessons from Patients with Chronic Kidney Disease

**DOI:** 10.3390/jcm11237130

**Published:** 2022-11-30

**Authors:** Eman Nagy, Mahmoud M. Sobh, Mohamed Abdalbary, Sherouk Elnagar, Rabab Elrefaey, Shimaa Shabaka, Nehal Elshabrawy, Rasha Shemies, Mona Tawfik, Cássia Gomes S. Santos, Fellype C. Barreto, Amr El-Husseini

**Affiliations:** 1 Mansoura Nephrology and Dialysis Unit, Mansoura University, Mansoura 35516, Egypt; 2 Department of Internal Medicine, Division of Nephrology, Federal University of Paraná, Curitiba 80060-00, PR, Brazil; 3 Division of Nephrology & Bone and Mineral Metabolism, University of Kentucky, Lexington, KY 40536-0298, USA

**Keywords:** low bone turnover, renal osteodystrophy, CKD–MBD, calcification, management

## Abstract

Renal osteodystrophy (ROD) is a common complication of end-stage kidney disease that often starts early with loss of kidney function, and it is considered an integral part in management of patients with chronic kidney disease (CKD). Adynamic bone (ADB) is characterized by suppressed bone formation, low cellularity, and thin osteoid seams. There is accumulating evidence supporting increasing prevalence of ADB, particularly in early CKD. Contemporarily, it is not very clear whether it represents a true disease, an adaptive mechanism to prevent bone resorption, or just a transitional stage. Several co-players are incriminated in its pathogenesis, such as age, diabetes mellitus, malnutrition, uremic milieu, and iatrogenic factors. In the present review, we will discuss the up-to-date knowledge of the ADB and focus on its impact on bone health, fracture risk, vascular calcification, and long-term survival. Moreover, we will emphasize the proper preventive and management strategies of ADB that are pivotal issues in managing patients with CKD. It is still unclear whether ADB is always a pathologic condition or whether it can represent an adaptive process to suppress bone resorption and further bone loss. In this article, we tried to discuss this hard topic based on the available limited information in patients with CKD. More studies are needed to be able to clearly address this frequent ROD finding.

## 1. Introduction

Chronic kidney disease (CKD) is a global public health epidemic comprising a major overwhelming threat to bone and mineral metabolism with a constellation of renal osteodystrophy (ROD) and cardiovascular disease known as chronic kidney disease–mineral bone disorder (CKD–MBD). Almost all patients with CKD–MBD have distinct abnormal bone pathology within the spectrum of ROD, including osteitis fibrosa, adynamic bone (ADB), osteomalacia, mixed lesions, and osteoporosis [1,2]. ADB is primarily characterized by decreased or absent bone formation along with low cellularity of both osteoblasts and osteoclasts as well as thin osteoid seams and minimal or no peritrabecular or marrow fibrosis [3]. Authors may use the terms ADB and low bone turnover (LBT) interchangeably; however, the term ADB, unlike LBT, does not include osteomalacia. ADB could be associated with a greater risk of vascular calcification (VC) and fractures substantiating detrimental outcomes [4,5,6]. It is not very well known whether all forms of ADB are truly pathological, and likewise milder degrees of ADB could be a compensatory mechanism to guard against bone loss. The history of ADB goes back to the 1970s when aluminum intoxication was first described in patients undergoing maintenance hemodialysis (HD) [7,8]. The intensive treatment of hyperparathyroidism with vigorous use of vitamin D analogs has continued to inflate the bubble of ADB [1]. Moreover, iatrogenic hypoparathyroidism after extensive parathyroidectomy for intractable secondary and tertiary hyperparathyroidism can lead to a severe form of ADB and aggravate cardiovascular calcification [6]. Of note, the prevalence of ADB has been further increasing in CKD with the expanding pool of the elderly and patients with diabetes mellitus [9,10]. The paucity of the gold-standard diagnosis by bone biopsies still challenges our understanding of ADB and impedes the elucidation of the underlying pathophysiologic mechanisms. Furthermore, using precision medicine to treat ROD based on the bone turnover status might improve bone health in patients with CKD. Future studies are expected to change the current perspectives and treatment of ADB.

## 2. Epidemiology

The term ROD describes the bone histomorphometry abnormalities in patients with CKD. It focuses on the changes in mineralization (M), bone turnover (T), and bone balance with subsequent bone volume (V) [11,12]. ROD comprises a distinctive spectrum of bone disease, including high and low bone turnover (HBT and LBT, respectively). It has long been believed that the most prevalent pattern of ROD in patients with advanced CKD and those on HD is osteitis fibrosa cystica induced by secondary hyperparathyroidism [13], whereas ADB is more prevalent in peritoneal-dialysis (PD) patients [14]. However, in the last two decades, there has been accumulating evidence supporting an ROD pattern shift, with more prevalence of LBT among patients with CKD [15]. In a large study conducted on 630 bone-biopsy specimens of patients on HD, investigators found LBT in 58% of patients (vs. 24% with HBT and 18% with normal bone pattern). Additionally, LBT was more predominant in the white than in the black population [1]. In a retrospective study that investigated the bone histomorphometry in 492 patients on dialysis in comparison to different bone biomarkers, the authors found a high percentage of LBT (59%) vs. HBT (17%) [16]. Barreto et al. also found an LBT pattern in 58 out of 97 (60%) patients on HD with inconsistent levels of parathormone (PTH), highlighting the poor specificity of PTH level in discriminating LBT and HBT [17]. The reason behind the changing pattern of ROD among patients on HD overtime could be related to aggressive management strategies targeting secondary hyperparathyroidism and changing patient characteristics [18]. 

Regarding CKD patients not on dialysis, much concern has been directed toward identifying the pattern and sequence of ROD in this group. Initial reports have demonstrated a high prevalence of ADB in early CKD stages [9,19]. Nevertheless, with progression of CKD, bone turnover moves towards a higher state because of a progressive increase in PTH that overcomes PTH resistance and bone-turnover–inhibitory factors [15]. ADB might be considered a transient state before HBT overcomes it with a progressive increase in PTH, which might be an adaptive mechanism to overcome low-turnover state and maintain normal bone formation rate while kidney function deteriorates [20]. Coen et al. assumed that ADB is a transitional stage before hyperparathyroid bone disease dominates [9]. Later, Barreto et al. described a higher prevalence of LBT in early-CKD patients (stage 2–3), whereas HBT was the predominant pattern in advanced CKD (stage 4–5) [19]. In a recent cross-sectional study, El-Husseini et al. demonstrated that 84% of patients with mild to moderate CKD had LBT. They also reported that abnormal bone quality and cardiovascular calcifications are predominant in these patients [20]. 

It is unknown whether LBT is a transitional state before development of HBT or is a separate entity in view of the lack of observational longitudinal studies. A prospective study demonstrated that bone turnover increases over time in patients with CKD stages 2–4. In this study, 87% of patients had LBT at baseline, and this percentage went down to 78% after 2 to 3 years of observation [21]. Table 1 summarizes the main bone histomorphometric studies in patients with CKD.

## 3. Pathogenesis

**Figure 1:**
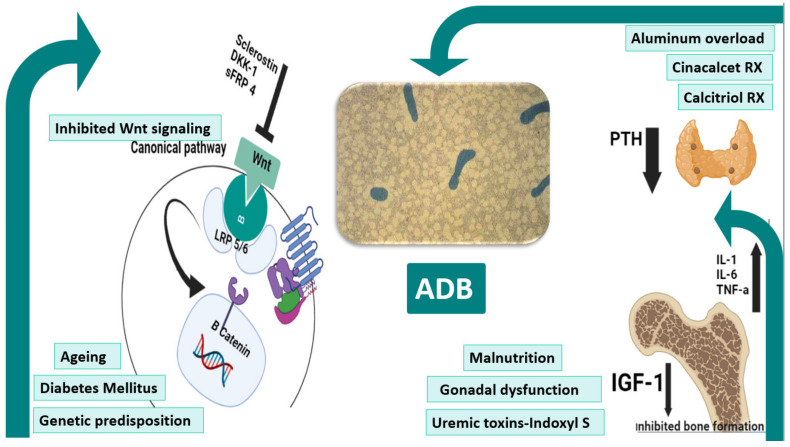
Pathogenesis of ADB. Ageing, diabetes, and genetic factors underlie the pathogenesis of ADB. Scelorstin, DKK1, and sFRP4 inhibits the extracellular binding of wnt to the Frz-LRP5/6 receptor complex blocking the B-catenin-mediated expression of target genes. Hypoparathyrodism precipitated by excessive treatment with cinacalcet and calcitriol therapies together with uremic milieus such as malnutrition, gonadal dysfunction, and uremic toxins all predispose to ADB. The central figure shows acellular bone with bone volume in a patient with ADB. ADB: adynamic bone, DKK-1: Dickkopf-related protein-1, IGF-1: insulin-like growth factor-1, IL-1: interleukin-1, IL-6: interleukin-6, LRP5/6: low-density lipoprotein receptor-related protein 5/6, PTH: parathyroid hormone, sFRP 4: secreted frizzled-related protein 4, TNF-α: tumor necrosis factor-α.

The pathophysiology of ADB is certainly multifactorial (Figure 1), comprising patient-related and iatrogenic factors on a predisposed genetic background. A state of imbalance between the low circulating levels of bone anabolic factors (e.g., insulin-like growth factor (IGF)-I) and the increased expression of bone-turnover–inhibitory factors, such as sclerostin and Dickkopf-related protein-1 (Dkk-1), largely predominates. This imbalance ultimately suppresses bone formation through repression of WNT/β-catenin signaling [18]. Moreover, uremic toxins may play a role in this setting. Uremic toxins are compounds that accumulate in the body as renal function declines, adversely affecting the function of multiple organs and systems. Indoxyl sulfate, a protein-bound uremic toxin (PBUT) generated by the gut microbiome from the metabolism of tryptophan, has been linked to LBT [15]. Experimental evidence suggests that the gut-derived toxin indoxyl sulfate aggravates LBT and inhibits osteoclast function and differentiation; however, further clinical studies would be more conclusive [31,32,33]. Of note, the circulating cytokines, for instance interleukin-1 (IL-1), IL-6, and tumor necrosis factor α (TNFα), have been supposed to directly impair bone-forming and -resorbing cells [34,35,36]. 

Patients on PD are more vulnerable to ADB due to high dialysate-calcium concentration [37] and possibly increased glucose load [38]. Aging, diabetes mellitus, malnutrition, and alcoholism have all been shown to be risk factors for the development of ADB [39]. Aluminum overload resulting from contamination of HD water and non-judicious use of aluminum-containing phosphate binders represented a major culprit a few decades ago [15]. In the 1980s, aluminum intoxication was the most prevalent cause of LBT in patients on dialysis [40]. Aluminum significantly decreases both osteoclast and osteoblast activities and causes defective mineralization [39,41]. Undoubtedly, the armamentarium emerged to control secondary hyperparathyroidism with vigorous use of oral calcium and vitamin D sterols, high dialysate-calcium concentrations, and surgical parathyroidectomy-induced hypoparathyroidism has extensively contributed to the growing prevalence of ADB [18,42,43]. Parathyroidectomy has been shown to cause a shift in bone turnover from high to low in conjunction with progressive VC [6].

Malnutrition may be another co-player in patients with advanced CKD, particularly in PD patients [44]. Malnutrition–inflammation complex syndrome is common in patients with CKD and associated with a higher incidence of LBT [45]. The harmful effect of protein-energy wasting on bone quality may be secondary to increased proinflammatory-cytokine release [35] and the predominance of the catabolic state [46]. These combined factors may induce atypical variants of ADB with suppressed bone formation and increased bone resorption that leads to robust bone loss [47]. Additionally, inflammatory cytokines suppress the PTH synthesis and release [48]. A Japanese study involving over 15,000 dialysis patients reported an increased odds ratio of low PTH (<60 pg/mL) levels in the presence of low serum albumin and urea nitrogen concentrations used as surrogate markers of malnutrition [49]. Similarly, Sanchez showed that low serum albumin was associated with ADB in 44 patients with chronic PD [44]. Garipov et al. demonstrated in a cohort of kidney-transplant recipients (*n* = 839) that malnutrition/inflammatory syndrome was associated with a higher risk of bone fractures [45].

Gonadal dysfunction is one of the key facets that is perceived to negatively impact bone formation. Decreases in serum testosterone in men and estradiol in women are well known to impair bone health [50]. However, we should keep in mind that absolute or relative hypoparathyroidism remains the key player among all other factors, with resistance to the bone-stimulating effect of PTH caused by down-regulation of PTH receptors [18]. In addition, low vitamin D levels cause suppressed vitamin D receptor (VDR) expression [51], which has been found to be crucial in blocking osteoblast apoptosis and promoting osteoblast differentiation and thus maintaining normal bone formation and mineralization [52,53]. Ultimately, in a uremic milieu, the pathogenesis of ADB is additionally complex and warrants further exploration. It is crucial to be vigilant and try to avoid under- or over-suppression of the parathyroid gland to maintain appropriate bone turnover. 

Furthermore, diabetes mellitus is associated with impaired bone quality with a higher relative risk of overall fracture [54]. Several bone histomorphometric studies in diabetic patients observed that ADB is the predominant type of diabetic osteodystrophy [55,56]. Various inhibitors of bone formation, such as sclerostin, lead to ADB in these patients [57,58,59]. Moreover, advanced glycation end-products (AGEs) can interfere with osteoblast and osteoclast activity, and its accumulation in bone matrix may have a negative effect on bone strength [60,61,62]. In addition, gastrointestinal hormonal disturbances, insulin deficiency, and IGF-I resistance are other contributing factors [63,64].

## 4. Impact of LBT on Bone Health/Fracture, Osteoporosis, and Mortality

Adynamic bone results in poor skeletal health, bone fragility, and diminished ability to restore damaged bone [39]. A crucial aspect of delayed remodeling is that it promotes more secondary mineralization, making the bone stiffer/tougher [65]. However, in the long term, over-mineralization can induce brittle bone that increases the risk of atypical fractures [66]. Moreover, suppression of bone turnover may cause microcracks, which are difficult to heal in the presence of low bone formation [67]. The uncoupling of bone turnover with a relative predominance of resorption may also contribute to bone loss in patients with ADB and CKD [18]. El-Husseini et al. previously found that the prevalence of LBT and HBT was comparable in osteoporotic patients [68]. Moreover, Barreto et al. demonstrated that the prevalence of low trabecular bone volume was relatively higher in patients with LBT compared to those with HBT [69]. Carbonara et al. reported that there was no significant difference in the prevalence of osteoporosis among patients with different types of ROD in another bone-histomorphometry study [28]. Despite initial reports not finding any fracture-risk difference based on ROD pattern [25,26], Hughes-Austin et al. recently revealed that patients with biomarker-defined LBT had an eight-fold higher risk of fracture [70]. 

Several studies have reported a J- or U-shaped association between PTH levels and mortality in patients on dialysis from different geographic areas [71,72,73,74,75,76,77]. Several studies concluded that low PTH levels, indicative of LBT, were associated with a higher risk of mortality [75,78]. In a subgroup analysis of 5387 patients on HD from the Dialysis Outcomes and Practice Patterns Study (DOPPS) phases 1–4, naïve of CKD–MBD treatment, PTH levels < 50 pg/mL were associated with 25% higher mortality in comparison to PTH levels between 150 and 300 pg/mL [77]. A Korean study reported that serum PTH < 150 pg/mL was an independent risk factor for infection-related mortality, compared to the target range of PTH 150–300 pg/mL in incident dialysis patients (*n* = 1260 HD; 511 PD) [74]. In the CORES study, which included 16,173 Latin American HD patients, once again PTH < 150 pg/mL was associated with all-cause and cardiovascular mortality [76]. Although the association between low PTH levels and mortality in different populations strengthens the clinical significance of ADB, it should be kept in mind that these studies were observational, preventing a causal effect from being established. Moreover, we cannot completely rule out the influence of residual confounders such as inflammation and malnutrition, which are commonly associated with low PTH levels and may also influence clinical outcomes.

However, studies investigating the association between biopsy-proven ADB and mortality are scanty. Data of the Brazilian Registry of Bone Biopsy (REBRABO) from patients with stage 3–5D CKD who underwent bone biopsy between 2015 and 2018 and were followed up on for up to 30 months did not find any association between hospitalization or mortality and neither the type of ROD nor the TMV classification [28]. The relatively short period of follow-up and the low number of events (56 hospitalizations, 14 deaths) might explain this negative result. The differences in mortality risk in patients with ADB might also be attributed to other confounders, such as VC, fractures, and the cause of LBT [79].

## 5. ADB as a Risk Factor for Vascular Calcification

Cardiovascular disease is considered the main cause of death in the CKD population [80]. VC is common in CKD in both pre-dialysis and dialysis patients, and it has been linked to high CKD-related cardiovascular mortality [81]. ADB leads to reduced bone capacity to buffer calcium and inability to handle an extra calcium load [82]. Experimental studies have demonstrated the crucial role of increased levels of calcium and phosphate and the importance of bone turnover in uremic VC [81,83]. Radiologically, VC may be presented as cardiovascular calcifications, chondrocalcinosis, or periarticular calcifications. In severe forms, large periarticular calcifications can cause well-defined, lobular, calcified masses around the joints called “tumoral calcification” [84]. Of note, ROD-associated VC affects mainly the vascular media as opposed to atherosclerotic calcification, which affects mainly the intima of the blood vessels [84].

Several clinical studies have investigated the possible association between LBT and VC in CKD. London et al. demonstrated in a cohort of 50 patients on HD that higher arterial-calcification scores, evaluated by ultrasonography and X-ray, were associated with biochemical and histomorphometric signs of ADB [5]. Similarly, in a prospective study in patients on HD, Barreto et al. demonstrated that LBT status was an independent predictor for coronary arterial calcification (CAC) progression [27]. In a study that included 207 patients on HD, CAC was associated with a significantly lower bone-formation rate [85]. Moreover, there was a negative association between parameters of bone formation, i.e., activation frequency and bone formation rate, and CAC scores in patients with LBT [85]. In PD patients, the only bone-biopsy-based study to date that evaluated the relationship between VC and ROD found no association between VC and bone histomorphometric parameters [86]. The small sample size (*n* = 41), the use of a semiquantitative method to evaluate VC (Kaupilla score), and the relatively low prevalence of VC (24.3%) may, at least partially, explain this unexpected result. In pre-dialysis patients, low bone-formation rate has been independently associated with VC, evaluated either by CT or by plain X-ray [4,87].

Calciphylaxis is a rare type of VC associated with a high mortality rate. Calciphylaxis involves the medial layer of small arteries and arterioles and, less frequently, the intimal layer and the interstitium of subcutaneous adipose tissue [88]. The occurrence of calciphylaxis in HD patients with biopsy-proven ADB [89,90] or biochemical signs of LBT has been reported [91]. Interestingly, a large European nationwide registry suggests that, contrary to ancient reports, uncontrolled hyperparathyroidism is not a key determinant of calciphylaxis [92].

Serum levels and bone expression of sclerostin increase with renal function decline, and it has been suggested that sclerostin may play a role in the pathogenesis of ADB [93,94]. Serum levels of sclerostin correlate inversely with bone turnover [29,86] and directly with VC [95,96]. More recently, in an elegant experimental study, Mace et al. found that calcified aorta from uremic rats secretes high amounts of sclerostin into the circulation, which could potentially inhibit Wnt signaling in bone and impair bone formation [97]. This striking finding indicates that (i) sclerostin might be an important messenger in bone–vessel crosstalk and (ii) there may be a bidirectional pathway between bone and vessel in which LBT may contribute to the development of VC and vice-versa [98]. The association between sclerostin and mortality in dialysis patients is controversial. Some studies have reported that high serum levels of sclerostin are associated with higher mortality rate [99,100,101], whereas others have pointed in the opposite direction [102,103]. These conflicting findings highlight the complexity of the relationship between bone, vascular disease, and mortality.

## 6. Variants of ADB

The term ADB might be misleading, as it may suggest that bone becomes completely static. However, bone resorption and formation occur uncoupled and at a slower pace, favoring in most cases the former, a scenario that may ultimately lead to bone loss over time [17]. 

Minimodeling is a process by which bone formation occurs in a quiescent bone area, i.e., in the absence of prior bone resorption. Although it is a rare phenomenon in normal individuals, it can continue throughout life and may be important to maintaining some degree of bone formation in certain conditions [104]. In an elegant study that analyzed bone biopsy from patients on dialysis, Ubara et al. found that minimodeling was significantly greater in patients with ADB than in patients with osteitis fibrosa and positively correlated with total bone volume [105]. Minimodeling was more pronounced in young patients and in those able to perform their daily activities [105]. Therefore, minimodeling might be an important mechanism to compensate for the absence of remodeling stimulated by PTH, contributing to mitigating the loss of bone integrity in ADB. In line with these findings, it has also been shown that minimodeling may also contribute to bone formation in other conditions characterized by low PTH levels, such as primary hypoparathyrodism and after parathyroidectomy [106]. It has also been reported that ADB might be associated with higher-than-expected osteoclast activity for the PTH levels [107,108].

## 7. Diagnosis

**Figure 2:**
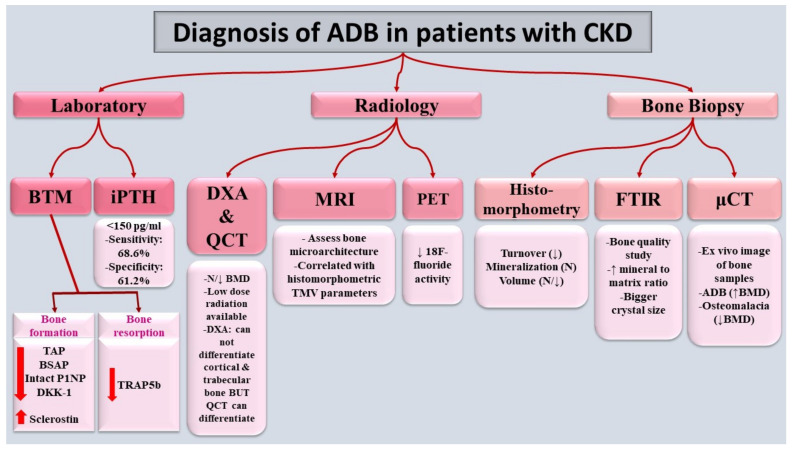
Diagnosis of ADB in patients with CKD. Diagnosis mainly relies on bone biopsy, gold standard, and reduced bone-formation and bone-resorption markers. ADB: adynamic bone, BSAP: bone-specific alkaline phosphatase, BMD: bone-mineral density, BTM: bone-turnover markers, CKD: chronic kidney disease, DKK-1: Dickkopf-related protein-1, DXA: Dual-energy X-ray absorptiometry, FTIR: Fourier-transform infrared spectroscopy, iPTH: intact parathyroid hormone, MRI: magnetic-resonance imaging, P1NP: procollagen type 1 N-terminal pro-peptide, PET: positron emission tomography, QCT: quantitative computed tomography, TAP: total alkaline phosphatase, TMV: turnover/mineralization/volume, TRAP 5B: tartrate-resistant acid phosphatase 5b, μCT: micro-computed tomography.

Despite bone biopsy being the gold-standard method for the diagnosis of ADB, non-invasive tools can help not only to diagnose ADB but also to follow up on the response to the pharmacological and non-pharmacological interventions (Figure 2).

### 7.1. Bone-Turnover Markers (BTMs)

BTMs provide mild-to-moderate precision evaluation of bone health in patients with CKD [109].

#### 7.1.1. PTH

Despite its limitations, intact or bio-intact PTH is the most widely used biomarker for CKD–MBD monitoring. LBT should be considered when iPTH is < 150 pg/mL in patients with advanced CKD [110]. Sensitivity and specificity of iPTH cutoff of less than 150 pg/mL for diagnosis of ADB are 68.6% and 61.2%, respectively [16]. However, the target iPTH levels in patients on dialysis (2–9-fold upper limit of normal) do not exclude the possibility of LBT [17]. 

#### 7.1.2. Bone-Formation Biomarkers

Despite both serum total and bone-specific alkaline phosphatase (TAP, BSAP) being biomarkers for osteoid formation, BSAP is more precise in discriminating the bone-turnover status in patients on dialysis [110,111].

Procollagen type 1 N-terminal pro-peptide (P1NP) and procollagen type 1 C-terminal pro peptide (P1CP) are indicators of collagen-synthesis rate [112,113]. However, in patients with CKD, only intact P1NP is reliable, as it is not affected by either glomerular-filtration rate (GFR) or dialysis [112,114,115]. Bone-derived osteocalcin is another bone-formation biomarker; however, its usage is limited in patients with advanced CKD because it is renally cleared [116]. Despite few studies having demonstrated a poor association between these biomarkers and bone-formation rate [117,118], others have found an association between high serum levels in these biomarkers and diminished cortical thickness and density, and increased porosity [119].

Sclerostin and Dkk-1 are soluble inhibitors of Wnt-signaling pathway, which is the main promotor for bone formation [120,121]. Studies found a significant association between high sclerostin levels and ADB [86,93,94,122]. Despite Cejka et al. not finding an association between serum Dkk-1 levels and bone-formation rate [93], Neto et al. found a positive association between low serum Dkk-1 levels and ADB [29].

#### 7.1.3. Bone-Resorption Biomarkers

Although carboxy-terminal crosslinking telopeptide of type 1 collagen (CTX) is the reference bone-resorption marker in a normal population, its use is limited in patients with CKD, as it is renally excreted [113,123]. On the other hand, tartrate-resistant acid phosphatase 5 b (TRAP5b) is unaffected by either renal function or dialysis, and patients with higher levels showed a faster rate of cortical-bone loss [124], as well as other bone-resorption and histomorphometric parameters [112,125].

The receptor activator of nuclear factor-κB (RANK)/receptor activator of nuclear factor-κB ligand (RANKL)/osteoprotegerin (OPG) system is responsible for the fine integration between osteoblasts and osteoclasts for bone formation and remodeling [114]. However, their utility as bone-resorption markers is questionable [122]. 

#### 7.1.4. Other Biomarkers

SIBLING (small integrin-binding ligand N-linked glycoprotein) and SIRT1 (Sirtuin 1) proteins are also regulatory proteins involved in bone remodeling and mineralization [114,126,127]. These novel regulators might be useful in the development of new treatment strategies for ROD [127].

### 7.2. Radiology

#### 7.2.1. Dual-Energy X-ray Absorptiometry (DXA) Scan and Quantitative Computed Tomography (QCT)

Patients with ADB may have either low or normal bone-mineral density (BMD) [84]. In patients with CKD, DXA scan has several limitations, including confounded results due to soft-tissue calcifications and osteoarthritis. [128]. On the other hand, QCT is less confounded by these abnormalities, and moreover, it discriminates trabecular from cortical bone. The major limitation of these diagnostic tools is the inability to identify the bone-turnover activity and the ROD type. Notably, DXA and QCT can be helpful in diagnosing asymptomatic vertebral fractures and cardiovascular calcifications that are not uncommon in patients with ADB [128,129]. Estimation of trabecular bone score by the DXA machine is a helpful non-invasive tool to evaluate the bone microarchitecture, which might reflect the bone quality and strength [128].

#### 7.2.2. High-Resolution Peripheral Quantitative Computed Tomography (HR-pQCT)

HR-pQCT assesses cortical and trabecular bone and precisely evaluates the microarchitecture [130]. Although a previous study reported that profound cortical-bone loss is suggestive of HBT [119], others reported no differences in most HR-pQCT parameters in groups with different iPTH levels [131,132].

#### 7.2.3. Positron Emission Tomography (PET) Scan

Fluorine 18–sodium fluoride (18F-NaF) is a bone-seeking tracer used in assessment of bone-turnover and osteoblast function [133]. A cross-sectional study in patients on dialysis reported a significant correlation between fluoride activity and histomorphometric parameters such as bone-formation rate, activation frequency, and osteoclast, osteoblast, and mineralized surfaces [134]. The sensitivity and specificity of this technique to discriminate LBT were 76% and 78%, respectively [134]. Moreover, another study appraised the diagnostic accuracy of ADB by PET scan compared to the gold-standard bone histomorphometry and revealed an area under the curve of 0.87 [129]. 

#### 7.2.4. Magnetic-Resonance Imaging (MRI)

MRI is a high-resolution technical modality that may aid in the assessment of bone microarchitecture. Sharma et al. reported a significant correlation between MRI indices and trabecular thickness and separation as well as mineralization and turnover parameters [135].

### 7.3. Bone Biopsy

Bone biopsy with mineralized-bone histology is the cornerstone to detecting different types of ROD [1,136]. Classification is mainly based on the turnover/mineralization/volume (TMV) system [12]. Turnover represents the process of skeletal remodeling, attained by both bone resorption and formation. It is assessed using bone-formation rate, activation frequency, and the number of osteoclasts and osteoblasts. Mineralization reflects the calcification of bone collagen and is assessed using osteoid volume, osteoid thickness, and dynamic, tetracycline-based evaluation of mineralization lag time and osteoid-maturation time. Lastly is volume, which reflects the amount of bone per unit [12,137]. In patients with ADB, bone histomorphometry reveals LBT with a reduced number of both osteoblasts and osteoclasts and low bone volume coupled with normal mineralization [12]. Of note, the presence of a thin osteoid is the main feature differentiating ADB from the other LBT-subgroup ‘‘osteomalacia’’ [138]. Interestingly, a different variant of ADB, characterized by low bone-formation rate in association with high osteoclastic resorption, was described. This variant might be a transitional phase between LBT and HBT [107,108]. Additionally, Misof et al. reported that bone-mineralization density distribution increases and the lacunar size of the osteocytes decreases in patients with ADB. This novel tool might be helpful to discriminate hyperparathyroid bone from ADB [139].

Although bone biopsy is the most precise tool to assess metabolic bone disease, it has some limitations, including high cost, lack of expertise, and limited patient acceptability [140]. Moreover, the wide discrepancy of the diagnostic cutoffs has led to significant differences in the classification of bone turnover [141]. Selection bias, age, gender, and ethnicity, in addition to the definition of normal-range differences, might explain the inconsistencies. Ultimately, it is better to unify the used cutoffs in order to properly estimate the prevalence and type of ROD to improve therapeutic decisions [141].

Micro-computed tomography (μCT) is a 3D high-resolution modality used to image small (millimeter-sized) bone samples using high amounts of radiation [142]. Notably, Pereira et al. demonstrated that μCT measurements might help to diagnose ROD types in children with end-stage kidney disease [143].

Fourier-transform infrared spectroscopy (FTIR) is a qualitative research tool to analyze mineral quality and bone-matrix properties in bone-biopsy specimens. Recovery of LBT might improve bone quality with a balanced mineral-to-matrix ratio and formation of smaller crystals. In addition, changes in crystal size correlated negatively with changes in trabecular separation, which indicates higher bone turnover [20,144].

## 8. Importance of Determining the Underlying Etiology of ADB

The underlying etiology of ADB dictates not only the natural history of ROD, but also the progression of cardiovascular disease and patient survival. For instance, management and outcome of ADB induced by anti-resorptives, vitamin D analogues, calcimimetics, and parathyroidectomy are probably different from ADB secondary to old age, diabetes mellitus, malnutrition, and chronic inflammation [145,146,147,148]. In iatrogenic cases of ADB without underlying systemic illnesses, the suppressed bone turnover might be well balanced and the osteoblast function could be relatively preserved [149,150,151,152]. On the other hand, diabetes and chronic inflammation lead to uncoupling of bone turnover that can induce robust bone loss [153,154]. This concept generated the following hypothesis: ADB might not be a real disease by itself, and the poor outcome could be confounded by the underlying disorder [42]. Moreover, the balanced forms of suppressed bone turnover might serve as an adaptive mechanism for bone preservation, as observed in hibernating bears [42,155,156,157]. This notion would pave the way for less restrict use of anti-resorptive medications in patients with osteoporosis [158]. However, one may make an argument, as the absence of clinical evidence of harm does not mean an absence of harm [159]. Furthermore, clinical trials did not stratify patients based on turnover status. Thus, a harmful effect in the adynamic group might be diluted by the beneficial effect in the HBT group. Finally, safety concerns were still observed in long-term follow-up after parathyroidectomy [6]. 

## 9. Prevention and Treatment of ADB in CKD

Despite the underlying complex pathophysiology of ADB, its management is based mainly on the avoidance of risk factors associated with reduction of bone turnover, such as aluminum exposure, over-suppression of PTH secretion due to calcium overload, administration of high doses of vitamin D analogs, and/or calcimimetics. Moreover, factors that may contribute to PTH resistance, such as hyperphosphatemia, malnutrition, inflammation, and progression of CKD, among others, should also be targeted [22,160].

### 9.1. Over-Suppression of PTH Secretion

#### 9.1.1. Vitamin D Analogs and Calcimimetics

The observed increase in the prevalence of ADB during the last few decades may be, at least partially, explained by the overtreatment of secondary hyperparathyroidism [161,162]. Calcitriol may directly suppress bone turnover by inducing osteoblast apoptosis independently of PTH levels [163]. Interestingly, paricalcitol, a synthetic vitamin D analog, seems to be associated with a lower risk of ADB compared with calcitriol, probably due to its higher selectivity to the VDR in the parathyroid glands and less calcemic effect [164,165]. Therefore, using vitamin D analogs and/or calcimimetics with caution to achieve the appropriate PTH levels, as well as reducing or, whenever necessary, withdrawing these medications when PTH is over-suppressed, are important approaches to avoid ADB [79,160]. Notably, the optimal PTH levels for patients with CKD G3a-G5 remains to be established, and the target range of PTH levels for CKD 5D proposed by international guidelines are not a guarantee of normal bone turnover [17,166]. Defining the optimal serum concentration of PTH across CKD stages, especially at an individual level, is still an unmet challenge.

From a practical point of view, we advocate regularly monitoring the serum levels of both PTH and biomarkers of bone turnover, such as TAP or BSAP, in order to assure adequate control of parathyroid function without excessive suppression of bone turnover. Moreover, as an increase in PTH levels is part of the adaptive response, due to its phosphaturic action and the bone resistance to this hormone, it seems reasonable to tolerate slight increases in PTH levels, particularly in the elderly and in diabetic patients with CKD in the pre-dialysis stage. We also encourage monitoring the PTH levels over time rather than preemptively starting therapies to suppress PTH secretion [166,167]. Similarly, when choosing the type of surgery to treat secondary hyperparathyroidism, it might be wise to consider gentle subtotal parathyroidectomy, as total parathyroidectomy is associated with a higher risk of postoperative hypoparathyroidism and, consequently, ADB [42].

#### 9.1.2. Calcium Overload

##### Calcium Intake

The KDOQI guideline recommends limits for calcium intake of 800–1000 mg/d (including dietary calcium, calcium supplements, and calcium-based phosphate binders) for adults with CKD stage 3–4. To maintain a neutral calcium balance, in CKD on dialysis calcium intake should be adjusted, taking into account the concomitant use of vitamin D analogs and calcimimetics, in order to avoid positive calcium balance [168]. Indeed, this approach may have beneficial effects on patients with CKD, as calcium overload has been linked to both ADB and VC [5]. Importantly, the reduced bone-buffer capacity in LBT states may contribute to ectopic calcification [42]. That said, it has been recommended to use non-calcium rather than calcium-containing phosphate binders in patients with ADB/low PTH levels, as this could help with relaxing PTH suppression and stimulating bone turnover [79,160]. Mathew et al. demonstrated, using an animal (LDL-receptor knockout mice) model of CKD, that sevelamer can decrease VC and ameliorate bone formation [83]. In the clinical setting, Barreto et al. demonstrated that lower trabecular-bone volume was associated with CAC development in patients on HD, while improvement in bone turnover was associated with lower CAC progression in patients with HBT and LBT irrespective of phosphate-binder choice (calcium-based vs. non-calcium-based). Conversely, Ferreira et al. showed that patients treated with sevelamer demonstrated an increase in bone formation when compared to patients treated with calcium carbonate [169]. A meta-analysis of randomized controlled trials (RCT) found that non-calcium-based phosphate binders were associated with a significantly higher bone-formation rate than calcium-based phosphate binders [170].

##### Dialysate Calcium

The concentration of calcium in the dialysate may also impact bone metabolism in patients on dialysis. Haris et al. demonstrated that low-calcium dialysate (2 mEq/L) for 16 months in patients on PD with biopsy-proven ADB was associated with lower levels of serum ionized calcium, fewer episodes of hypercalcemia, and an increase in PTH levels, leading to normalization of bone formation [37]. Despite the standard dialysate calcium in PD solutions being 3.5 mEq/L, it has been suggested to lower dialysate calcium in patients with LBT [171]. Moreover, the KDIGO guidelines recommend dialysate calcium fluid of 2.5 mEq/L, as several studies on HD patients with biochemical signs of ADB have shown that using 2.5 mEq/L dialysate calcium may increase serum levels of PTH and biomarkers of bone turnover, such as BSAP and TRAP-5b [172,173,174]. Importantly, reduction of dialysate calcium exposure may not only improve ADB but also slow down the progression of VC [79].

### 9.2. Antiresorptives

The induction or aggravation of LBT is a major safety concern regarding antiresorptive use in patients with CKD, particularly because the uremia may be an essential modifier of the bone response to these agents. This is more worrisome with long-term use or high doses of certain bisphosphonates that may lead to bone becoming more brittle, less tough, and more likely to fracture [175]. In this context, compared to placebo, long-term use of denosumab in post-menopausal females was associated with normal bone histology, higher matrix mineralization, less bone remodeling, and lower mineralization heterogeneity, while maintaining the benefit of fracture risk [176,177]. Although there is permanent suppression of bone turnover for the duration of therapy with bisphosphonate and even thereafter (long skeletal half-life), denosumab leads to an early and profound decline in bone turnover after each dose, which thereafter partially recovers before the subsequent administration. Whether these variations in pharmacodynamics translate to various risks of atypical fractures remains to be determined [176]. Although bisphosphonates are retained by the skeleton and only slowly released, it is yet unclear whether they could have unfavorable effects on bone remodeling in CKD. Alendronate has been demonstrated to significantly increase femoral neck-and-spine BMD and decrease the risk of fractures in women with advanced CKD followed for up to 4 years, without increasing the incidence of adverse events [178]. Clinical studies evaluating the effects of antiresorptive medications on vascular outcomes in patients with CKD are scanty. In a recent RCT, treatment with denosumab or alendronate for 12 months improved BMD and did not affect the health of blood vessels (including scores of VC) in patients on dialysis [145]. Several studies showed that the fracture risk increases after discontinuation of denosumab in osteoporotic women with normal kidney function [179,180,181,182,183,184]. Studies addressing this concern in CKD are lacking. Of note, Mazurenko and Feofanova reported a case of severe bone loss and multiple fractures after denosumab discontinuation in a diabetic woman on HD [185]. Finally, as bisphosphonates are the most affordable anti-osteoporotic medications [186], it seems reasonable to consider their use in patients with CKD in a case-by-case basis, taking into consideration bone turnover. Studies evaluating the efficacy and safety of antiresorptives in patients with advanced CKD are urgently required.

### 9.3. Malnutrition–Inflammation Syndrome

Obesity and higher body mass index are directly associated with PTH levels in pre-dialysis CKD patients, independently of GFR, given further support of the link between nutritional status and CKD–MBD [187]. Therefore, one may hypothesize that improvement of the nutritional status and the inflammatory profile of patients with CKD may be interesting strategies to increase bone turnover. Clinical studies have suggested that blocking inflammatory cytokines may have a positive effect on BMD. The inhibitory effect on pro-inflammatory cytokines related to bisphosphonates may be a factor in their beneficial impact on bone mass [42,45].

### 9.4. Uremic Toxins

Therapeutic strategies to reduce intestinal generation or to increase removal by dialysis may be interesting approaches to improving bone turnover. Indeed, an oral dose of an intestinal adsorbent to reduce uremic toxins (AST-120) resulted in increased bone formation in an animal model of CKD [188]. Despite this encouraging finding, no clinical trial has demonstrated the impact of AST-120 on bone metabolism in uremic patients so far. More recent studies suggest that oral charcoal adsorbent, additive charcoal in the dialysate, and mixed-matrix-membrane hollow fiber, in conjunction with conventional dialysis therapy as well as the use of binding competitors during HD, may enhance the removal of PBUTs, an effect that might help to improve bone turnover [189]. Finally, using an antagonist of the aryl hydrocarbon receptor, a ligand-activated transcription factor that controls the toxic consequences of uremic toxins, might be a potential additional strategy for preventing and treating ADB in the future [33]. 

### 9.5. Drugs Used for Potential Treatment of ADB

Bone builders or osteo-anabolics improve bone formation and BMD. Moreover, they decrease fracture risk in patients with age-related kidney dysfunction without real ROD [190]. However, the long-term efficacy of these medications for patients with CKD remains unknown [191]. Teriparatide showed efficacy in reducing the risk of fracture and improving vertebral and femoral neck BMD [191]. A recent study concluded that treatment with recombinant parathyroid hormone (1–84) in patients with chronic hypoparathyroidism over 5 years was associated with stable GFR compared to another untreated cohort in whom GFR declined by 1.67 mL/min/1.73 m^2^ per year [192]. Abaloparatide, a fragment of parathyroid hormone-related peptide, stimulates bone formation and is approved as osteo-anabolic therapy in postmenopausal women with osteoporosis [193]. Studies have suggested the safety and efficacy of its use in patients with mild to moderate CKD [194,195]. Romosozumab, sclerostin-humanized monoclonal antibody, acts as an anabolic and anti-resorptive concurrently. Romosozumab studies in patients with advanced CKD are limited, as there was a cardiovascular concern that might be related to increased vascular calcification with its usage [196]. Miller et al. reported on its efficacy in patients with mild to moderate CKD [197]. Moreover, two recent studies demonstrated one-year romosozumab cardiovascular safety in Japanese HD patients [198,199].

Patients with ADB should be followed up on longitudinally to monitor their progress and the response to pharmacological and non-pharmacological interventions. The use of BTMs to observe the changes over time is helpful, particularly in patients who are treated with anti-osteoporotic medications. Moreover, the use of a DXA scan and other imaging modalities is helpful to check the response to these various medications. Notably, after completing a year or two of a bone anabolic course, further treatment should rely upon the response to the intervention and the present bone-turnover status. Repeated bone biopsy might be warranted in patients who do not respond appropriately to therapies or those whose bone turnover is difficult to assess non-invasively.

Table 2 summarizes the potential osteo-anabolic drugs for the treatment of ADB and their main respective studies.

## 10. Conclusions

ADB prevalence has been increasing in CKD, including in dialysis patients. It involves multiple pathogenetic mechanisms and risk factors. Its diagnosis depends mainly on bone biopsy in addition to non-invasive biomarkers. It is still unknown whether all cases of ADB are maladaptive or whether it can be adaptive/compensatory in certain situations. Management of ADB relies generally on prevention and treatment of its risk factors as well as use of osteo-anabolic medications.

## Figures and Tables

**Table 1:** Summary of the main bone histomorphometric studies in patients with CKD.

Study	Patient Population	Finding
**1. Malluche et al.** **1976** [22]	50 patients in different stages of CKD (GFR 6–80 mL/min/1.73 m^2^)	Prevalence of woven osteoid, as expression of osteitis fibrosa, was increasing with decreasing GFR. Osteoclastic surface resorption was abnormally high with GFR < 50. Mineralization defect was present only in patients with GFR < 40 mL/min/1.73 m^2^.
**2. Hutchison et al.** **1993** [13]	30 ESKD on CAPD	Osteitis fibrosa was the most common histological diagnosis (50%). ADB was found in 27%, mixed ROD in 13%, and osteomalacia in 7% of patients.
**3. Sherrard et al.** **1993** [14]	259 HD and PD patients	In PD patients 66% had LBT, whereas in HD patients 62% had HBT.Osteomalacia was the least common pattern (4% of all patients).
**4. Hernandez et al.** **1994** [23]	92 pre-dialysis patients with GFR < 10 mL/min/1.73 m^2^	ADB reported in 32%. Stainable bone aluminum surface was < 3% in all patients with ADB.
**5. Torres et al.** **1995** [24]	119 unselected CKD5: 38 were pre-dialysis, 49 on HD, and 32 on CAPD	ADB was a common finding (48%, 32%, and 48% in pre-dialysis, HD, and CAPD, respectively).
**6. Monier-Faugere and Malluche** **1996** [25]	2248 bone biopsies from ESKD patients on dialysis	HBT in 24.5%, mixed ROD in 52.9%, osteomalacia in 6.2%, and ADB in 16.4%. Patients on CAPD exhibited more histological signs of ADB.
**7. Coen et al.** **1996** [9]	76 pre-dialysis CKD patients, mean GFR 20 mL/min per 1.73 m^2^	Normal bone histology in 13%, ADB in 12% (all negative for aluminum staining), mixed ROD in 63%, predominant osteomalacia in 9%, and predominant HBT in 3%. Patients with ADB had a less severe degree of CKD.
**8. Araújo et al.** **2003** [26]	2507 dialysis patients who underwent bone biopsies (1985–2001) for diagnostic purposes.	Comparing the 1980s to the 1990s, the prevalence of patients with HBT increased from 32.3% to 44.0%, whereas aluminum overload decreased from 61.3% to 42.4%. Osteomalacia decreased whereas the prevalence of ADB increased from 15.7% to 20.4%.
**9. Barreto et al.** **2008** [27]	38 bone biopsies from HD patients	Baseline bone biopsies showed HBT in 37% and LBT in 63%; after 1 year of sevelamer or calcium acetate HBT was reported in 39% and LBT in 61% of patients.
**10. Malluche et al.** **2011** [1]	630 dialysis patients	LBT was diagnosed in 58%, normal turnover in 18%, and HBT in 24% of patients. With 3.5 mEq/L of dialyzed calcium, there were more LBT patients than with 2.5 mEq/L. White patients exhibited predominantly LBT, whereas HBT was the prominent feature in black patients.
**11. Barreto et al.** **2014** [19]	49 pre-dialysis CKD stages 2–5 patients	Patients at CKD stages 2–3 presented remarkable LBT. In comparison to patients with CKD stages 2 and 3, patients with CKD stages 4–5 showed higher osteoid volume, osteoblast and osteoclast surface, bone fibrosis, and BFR, as well as a lower MLT.
**12. Sprague et al.** **2016** [16]	492 dialysis patients	LBT in 59% of patients, HBT in 17% of patients, and 24% had normal turnover.
**13. Carbonara et al.** **2020** [28]	260 CKD–MBD stage 3–5D patients	HBT, mixed ROD, ADB, osteomalacia, osteoporosis, and aluminum accumulation were detected in 33%, 17%, 10%, 4%, 30%, and 25% of patients, respectively.
**14. Neto et al. ** **2021** [29]	56 patients with CKD stages 3–4.	ADB in 38%, HBT in 21%, and mixed ROD in 2% of patients, whereas 41% had normal bone histology.
**15. Jørgensen et al.** **2022** [30]	199 kidney-transplant candidates and recipients	Bone turnover was low in 17%, normal in 55%, and high in 29% of patients.
**16. El-Husseini et al.** **2022** [20]	32 CKD stage 2–4 patients with no specific bone-biopsy indication	84% had LBT, 6% had HBT, and 10% had normal turnover. LBT with abnormal bone quality was predominant in early CKD stages.

GFR: glomerular-filtration rate, ROD: renal osteodystrophy, ADB: adynamic bone, LBT: low bone turnover, HBT: high bone turnover, HD: hemodialysis, PD: peritoneal dialysis, ESKD: end-stage kidney disease, BFR: bone formation rate, MLT: mineralization lag time.

**Table 2:** Osteo-anabolics used for treatment of ADB.

Drugs	Mechanism of Action	Main Studies	Results
Teriparatide (PTH 1–34)	–A recombinant form of PTH, consisting of amino acids 1–34 that bind to PTH type 1 receptor stimulating osteoblast activity	Mitsopoulos et al.: 9 hemodialysis patients; 48 weeks of therapy [200].	With teriparatide: –Improvement in BMD (femoral neck: 2.7%; lumbar: 4.9%).
		Cejka et al.: 7 patients with ADB, 6-month therapy [201].	With teriparatide: –Improvement in BMD at lumbar spine. –No changes in BMD at femoral neck, bone-turnover markers, or CAC.
		Sumida et al.: 22 patients on dialysis; 56.5 μg dose once-weekly for 48 weeks [202].	With teriparatide: –High rate of discontinuation (50%) due to transient hypotension. –Improvement of BMD at lumbar spine by 3.3% and 3.0% at weeks 24 and 48, respectively. –No change in the BMD at femoral neck and distal radius.
Abaloparatide	–Fragment of parathyroid hormone-related peptide	Miller et al.: ACTIVE was phase 3, double blinded, RCT included 1645 postmenopausal women who received SC 80 μg abaloparatide or placebo daily [195].	With abaloparatide: Improvement in BMD at 1.5 years: –At total hip by 4.18%. –At femoral neck by 3.6%. –At lumber spine by 11.2%.
		Bilezikian et al.: post hoc analysis of ACTIVE to evaluate safety and efficacy of abaloparatide in patients with different kidney functions [194].	With abaloparatide: Improvement in BMD at 1.5 years: –At lumbar spine by 9.91% in patients with eGFR < 60 mL/min. –At femoral neck by 3.06% in patients with eGFR < 60 mL/min.
Romosozumab	–Sclerostin-humanized monoclonal antibody –Has anabolic properties	Miller et al.: post hoc analysis of FRAME and ARCH trials. FRAME included 7147 osteoporotic postmenopausal women who received 210 mg SC romosozumab or monthly placebo and ARCH (received 210 mg SC romosozumab monthly or 70 mg oral alendronate weekly) enrolled 4077 postmenopausal females with osteoporosis and fragility fractures [197].	With romosozumab In FRAME: Improvement in BMD at 1 year: –At lumbar spine by 13% and 10.9% in patients with mild and moderate CKD, respectively. –At total hip by 5.9% and 5.2% in patients with mild and moderate CKD, respectively. –At femoral neck by 5.3% and 4.6% in patients with mild and moderate CKD, respectively. In ARCH: Improvement in BMD at 1 year: –At lumbar spine by 8.8% and 8.1% in patients with mild and moderate CKD, respectively. –At total hip by 3.2% and 3% in patients with mild and moderate CKD, respectively. –At femoral neck by 3.2% and 2.7% in patients with mild and moderate CKD, respectively.
		Sato et al.: included 76 HD patients with high risk of fractures who received 210 mg SC romosozumab monthly and 55 HD untreated patients [199].	With romosozumab: –Improvement in BMD at lumber spine at 1 year by 15.3%. –Improvement in BMD at femoral neck at 1 year by 7.2%.
Ronacaleret	–Calcium-sensing receptor antagonist –Calcilytic –Increases endogenous production of PTH	Fitzpatrick et al.: placebo-controlled, dose-ranging trial. 569 women with post-menopausal osteoporosis, teriparatide 20 µg SC once daily or 100 mg, 200 mg, 300 mg, or 400 mg ronacaleret once daily; 70 mg alendronate once weekly; or matching placebos in a double-blind fashion [203].	–Improvement in spine integral vBMD (0.49% to 3.9%). –Improvement in trabecular vBMD (1.8% to 13.3%). –Non-dose-dependent decrease (1.79%) in integral vBMD at proximal femur.

ACTIVE: The Abaloparatide Comparator Trial in Vertebral Endpoints, ARCH: Active-Controlled Fracture Study in Postmenopausal Women with Osteoporosis at High Risk, BMD: bone-mineral density, CAC: coronary-artery calcification, CKD: chronic kidney disease, eGFR: estimated glomerular-filtration rate, FRAME: Fracture Study in Postmenopausal Women with Osteoporosis, HD: hemodialysis, PTH: parathyroid hormone, RCT: randomized clinical trial, SC: subcutaneous, vBMD: volumetric bone-mineral density.

## Data Availability

Not applicable.
